# SpatialFlux: an R package for distance gradient analysis in spatial transcriptomics

**DOI:** 10.1093/bioinformatics/btag543

**Published:** 2026-07-23

**Authors:** Dimitri Sokolowskei, Alexander J Trostle, Achira Shah, Robert J Tower

**Affiliations:** Department of Surgery, University of Texas Southwestern Medical Center, Dallas, TX, United States; Department of Surgery, University of Texas Southwestern Medical Center, Dallas, TX, United States; Department of Surgery, University of Texas Southwestern Medical Center, Dallas, TX, United States; Department of Surgery, University of Texas Southwestern Medical Center, Dallas, TX, United States

## Abstract

**Summary:**

Spatial transcriptomics (ST) data analysis and visualization face several challenges due to low sampling, diversity of tissue morphology and high drop-out inherent to the technique. New analysis methods are needed to overcome these challenges and promote continued biological discoveries. To overcome these constraints, we herein describe *SpatialFlux*, an R package developed to perform reference-based distance gradient analysis. *SpatialFlux* allows the identification and comprehensive visualization, in either an unbiased or biased manner, of differentially expressed genes and pathways across multiple ST tissues sections and along various axes, thus overcoming many inherent ST limitations and supporting continued biological discovery.

**Availability:**

*SpatialFlux* package source code and vignette are freely available on Github (https://github.com/towerlab/SpatialFlux) and Zenodo (https://zenodo.org/records/21039284).

## 1 Introduction

Spatial transcriptomics (ST) is a powerful and widely used method to assess gene expression in preserved tissue section (Tian *et al.* 2023, Sokolowskei *et al.* 2026). ST has provided critical insights into tissue diversity and composition, cellular processes and underlying mechanisms in health and disease (Williams *et al.* 2022, Li *et al.* 2025). However, substantial bottlenecks regarding ST data analysis and visualization currently exist, hampering its full potential. For instance, ST’s low sampling size and morphological discrepancies between tissues compromise statistical power and hinder reliable comparisons between different experimental groups. Moreover, interpretable, and comprehensive, spatially aware gene expression patterns and pathway visualization approaches are needed to interrogate complex biological processes within heterogeneous tissues.

Previously, we proposed a reference-based gradient distance analysis for investigating individual genes or pathways along a distance axis from spatial spots used as reference points (Tower *et al.* 2021). This approach relied on the establishment of an arbitrary set of spatial spots as refence spots followed by the calculation of the distance to all other targeted spots relative to the reference using Euclidean distances, thus enabling a gene expression assessment in a gradient-distance manner. This gradient-distance analysis strategy minimizes tissue architecture differences between samples by averaging gene expression across the distance axis, helping to obtain generalizable gene and pathways expression gradient across replicates and experimental conditions, combining multiple, morphologically distinct samples together to increase sample size, reducing noise and increasing statistical power.

Since then, this approach has been used to study traumatic injury (Tower *et al.* 2022a), the bone marrow microenvironment (Xiao *et al.* 2023), tissue regeneration (Tower *et al.* 2022b) and bone repair (Rios *et al.* 2024). Nevertheless, our approach lacked a cohesive, integrated format and ease-of-use for dissemination to the research community. Here, we present *SpatialFlux*, an R programming package that minimizes inherent ST data analysis limitations while providing an easy-to-use, reproducible, and comprehensive toolkit to investigate gene and pathway expression using spatial gradient distance for interrogating biological questions using spatial transcriptomics data.

## 2 Methods

**Figure 1:**
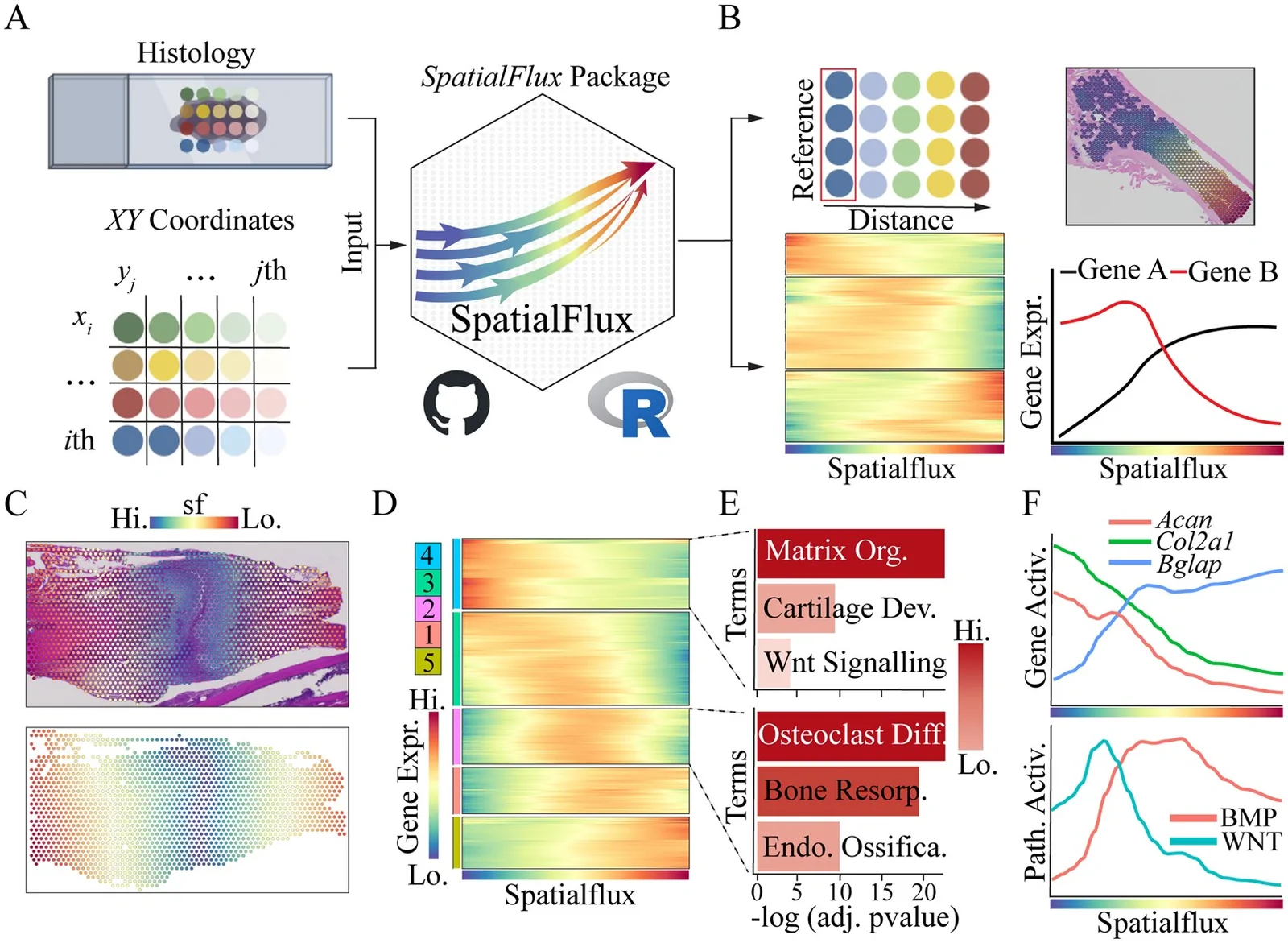
*SpatialFlux* package workflow and real-word dataset application. (A) Spatial transcriptomics *xy* coordinates from histology sections are used as input for the package. (B) Visualization approaches after selecting spatial spots as references points. (C) Distance analysis of all histology spots relative to reference (fracture site). (D) Heatmap showing gene clusters showing peak expression at different positions along the distance-gradient axis. (E) Gene ontology of enriched terms from genes showing peak expression nearest to and medium distance from the reference line. (F) Biased gene and pathway expression activity across SpatialFlux distance-gradient.

*SpatialFlux* is an R programming package applicable to 10× genomics Visium/VisiumHD-based (Ståhl *et al.* 2016, Oliveira *et al.* 2025) ST data and employs *Seurat* package object data format (Hao *et al.* 2024) as input. *SpatialFlux* makes use of the respective two-dimensional plane coordinates (*x*, *y*) representing spatial spots localization across the tissue histology for subsequent analysis (Fig. 1A). References are selected using the following possible strategies: (1) Individual spatial spot(s) selection using the package’s build-in interactive Shiny application, (2) spatial spot selection based on cluster identity, or (3) manually drawn reference lines using imaging analysis software such as Fiji/ImageJ (Schindelin *et al.* 2012). A full and detailed reference selection strategies walkthrough can be found in the package vignette (https://towerlab.github.io/SpatialFlux/). The distance between each reference point and all selected spatial spots can be calculated using Euclidean distance equation (1) as follows:


(1) $$d(x,\;y)\;=\;\sqrt{{\left(x_{i}\;-\;y_{i}\right)}^{2}\;+\;{\left(x_{j}\;-\;y_{j}\right)}^{2}}$$

Where, *x* = (*x_i_, x_j_*) and *y =* (*y_i_*, *y_j_*) represents the reference points and a given set of spatial spots of the *i*th row and *j*th column spatial spots, respectively.

Following Euclidean distance calculations, the minimum distance between each target spatial spot and the nearest reference point, referred to here as *spatialvalues*, is stored in the Seurat object metadata and used for preliminary gradient distance assessment. Unbiased detection of genes whose expression correlates with the distance from the selected references is calculated by substituting *spatialvalues* into a new object generated using Monocle3 (Trapnell *et al.* 2014, Cao *et al.* 2019) and using the internal Monocle3 differential gene expression function. Furthermore, the package provides options for individual genes and/or module score pathway visualization across the established distance axis enabling exploratory or biased gene expression analysis within a sample (Fig. 1B).

## 3 Results

In order to present *SpatialFlux* package usage, data analysis and visualization, we made use of a publicly available 10× Visium spatial transcriptomics dataset of a murine tibial fracture (Rios *et al.* 2024). To investigate underlying gene expression signatures within the mouse fracture site, spatial spots along the fracture site were manually selected (Fig. 1C). Euclidean distances between each spatial spot and the nearest reference spot were calculated for each individual image/slice, thus generating a distance gradient visualization, where the gradient colors represent the absolute distance of individual spots from the reference (Fig. 1C). Substituting *spatialvalues* into a Monocle3 formatted object and running differential gene expression analysis, combined with hierarchical clustering, identified 5 differentially expressed gene clusters with peak expression at unique distances from the fracture gap (Fig. 1D).

To investigate the possible biological role of the differentially expressed genes unbiasedly identified, each distance-associated gene cluster was used as input for gene ontology analysis. Genes upregulated near the fracture site (Fig. 1D, top) were enriched with terms associated with matrix organization, cartilage development and morphogenetic signaling, while genes expressed distant from the fracture gap (Fig. 1D, bottom) showed terms mainly related to bone cellular activity and mineralization (Fig. 1E). These results highlight major differences in gene expression landscape across different regions and cell types of a traumatic injury model, identified through unbiased means and corroborated by previous studies (Julien *et al.* 2021, Perrin *et al.* 2024). This same package workflow also allows the targeted assessment of known markers. Moving from high to low proximity from the fracture plane showed a rapid decline of cartilage-associated genes, including *Acan* and *Col2a1*, and a concomitant upregulation of the osteogenic gene *Bglap* as the callus histologically transitions from cartilage to bone (Fig. 1F). Moreover, *SpatialFlux* was able to draw comparable results between the fracture replicates and displayed a higher number of differentially regulated genes identified when using the combined replicates for gradient-distance analysis (Fig. S1, available as supplementary data at *Bioinformatics* online). This transition from chondrogenic to osteogenic genes coincided with the upregulation of early and late, pro-osteogenic morphogenetic pathways WNT and BMP, demonstrating the package’s capabilities in obtaining, visualizing and recapitulating underlying biological insights in complex biological processes and tissue environments.

To further validate *SpatialFlux* analytical capabilities, gradient-distance analysis was conducted to assess the more spatially refined liver zonation (Nikopoulou *et al.* 2023). By establishing spots based on the canonical periportal marker *Cyp2e1* as reference, our package was able to fully recapitulate core signaling gradient between pericentral to periportal liver zonation (Fig. S2, available as supplementary data at *Bioinformatics* online) supporting the tool capacity to assess spatially restricted gene expression profiles in highly complex tissue architectures at both coarse and fine tissue resolutions.

## 4 Conclusions

*SpatialFlux* provides users with an accessible, easy-to-use, integrated toolkit for sequencing-based ST data aimed at exploratory and targeted data analysis. In contrast to similar tools (Larsson *et al.* 2023, Kueckelhaus *et al.* 2024) developed after our initial concept description (Tower *et al.* 2021), *SpatialFlux* dispenses with the need for new object data structure initialization and handling or image alignment, permitting full gradient distance calculation across any histology coordinate. Additionally, *SpatialFlux* allows both biased or unbiased gene expression analysis across gradients, thus overcoming many analytical limitations seen in other tools (Feng *et al.* 2023, Larsson *et al.* 2023, Kueckelhaus *et al.* 2024, Chen *et al.* 2025) (Table S1, available as supplementary data at *Bioinformatics* online). While *SpatialFlux* was designed to support the widely adopted 10× Visium protocols, its basic functions have the potential to support any ST protocols containing gene expression spots and their respective *XY* coordinates. Using a distance-gradient principle, *SpatialFlux* allows comprehensive, spatially aware gene expression analysis underlying biological processes within any arbitrary histology segment. Further, *SpatialFlux* offers the ability to concomitate distance values from multiple samples, aligning spatial spots taken from morphologically unique histological sections onto a single *x*-axis, thereby increasing statistical power and removing biological noise, improving data generalization and biological discovery. The ease of use and diverse functionality of this package allows *SpatialFlux* to be used across multiple fields and will hopefully bring ST into the mainstream by more users.

## Author contributions

Dimitri Sokolowskei (Conceptualization [Equal], Data curation [Lead], Formal analysis [Lead], Investigation [Lead], Methodology [Lead], Software [Lead], Writing—original draft [Lead], Writing—review & editing [Lead]), Alex J. Trostle (Methodology [Supporting], Software [Supporting], Validation [Supporting], Visualization [Supporting]), and Achira Shah (Software [Supporting], Validation [Supporting]), and Robert J. Tower (Conceptualization [Equal], Funding acquisition [Lead], Project administration [Lead], Supervision [Lead], Writing—review & editing [Supporting])

## Supplementary Material

btag543_Supplementary_Data

## Data Availability

The spatial transcriptomics data used can be found at GEO: GSE218046.
