# Supplementary material for: SpatialFlux: an R package for distance gradient analysis in spatial transcriptomics
Source: Bioinformatics. 2026 Jul 23;42(8):btag543. doi: 10.1093/bioinformatics/btag543 (PMC13430652; doi:10.1093/bioinformatics/btag543)
Supplement: btag543_Supplementary_Data [file btag543_supplementary_data.zip › supplementary_materials.pdf]

Supplementary Materials

	SpatialFlux	Giotto (Chen et al., 2025)	SPATA2 (Kueckelhaus et al., 2024)	SPIAT (Feng et al., 2023)	Semla (Larsson et al., 2023)
Programming language	R	R	R	R	R
Distance Calculation	x	o	x	x	x
Biased gene expression	x	o	x	o	x
Unbiased gene expression	x	o	x	o	o
Reference selection	Multiple selection	o	Area selection	Individual spots selection	Angle and cluster selection
Protocol support	10x Visium	Multimodal	Multimodal	Multimodal (proteomics only)	10x Visium
Resolution	Subcellular	Subcellular	Subcellular	Subcellular	Subcellular
Specific object initialization	o	x	x	x	x

Table S1. ***SpatialFlux* package features comparisons against similar tools**. x = Feature present; o = feature not present.

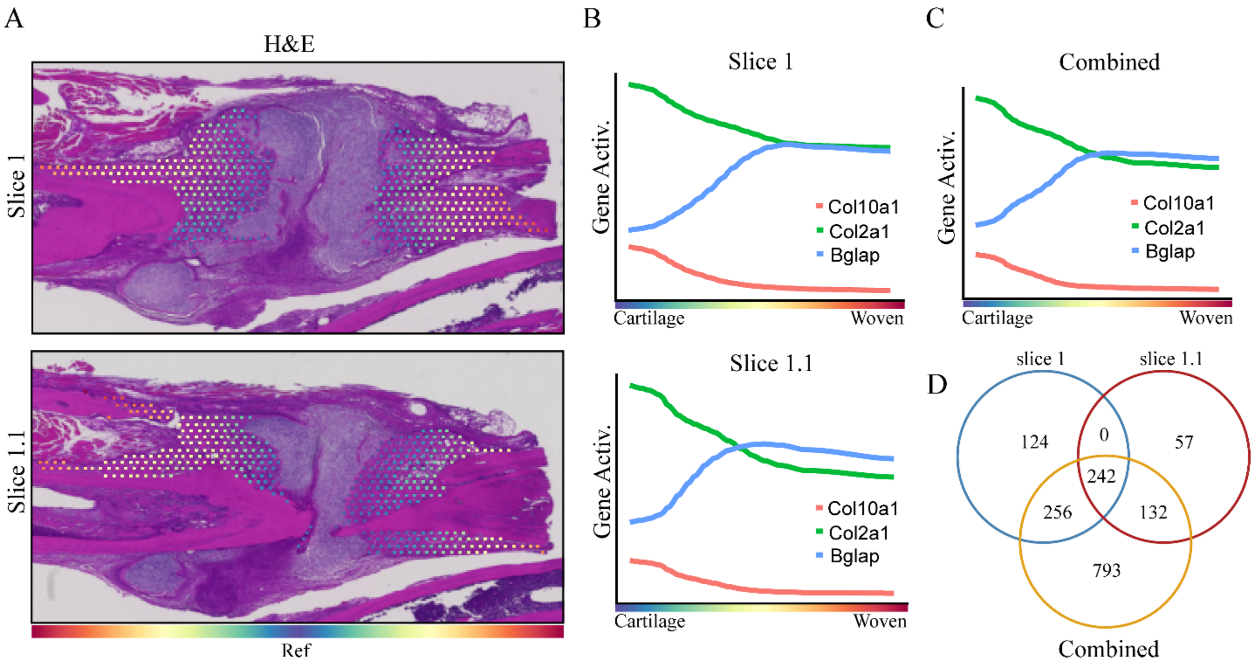

Figure S1 ***SpatialFlux* minimizes tissue morphological differences and increase gene expression statistical power**. (**A**) Gradient-distance analysis of murine tibia fracture model from cartilage to woven bone between replicates. (**B**) Curve plots of biased gene expression analysis across distance axis. (**C**) Biased gene expression curve plots of integrated sample replicates along the distance axis. (**D**) Venn diagram of differentially expression genes between each individual and integrated replicates highlighting packages potential to improve statistical power.

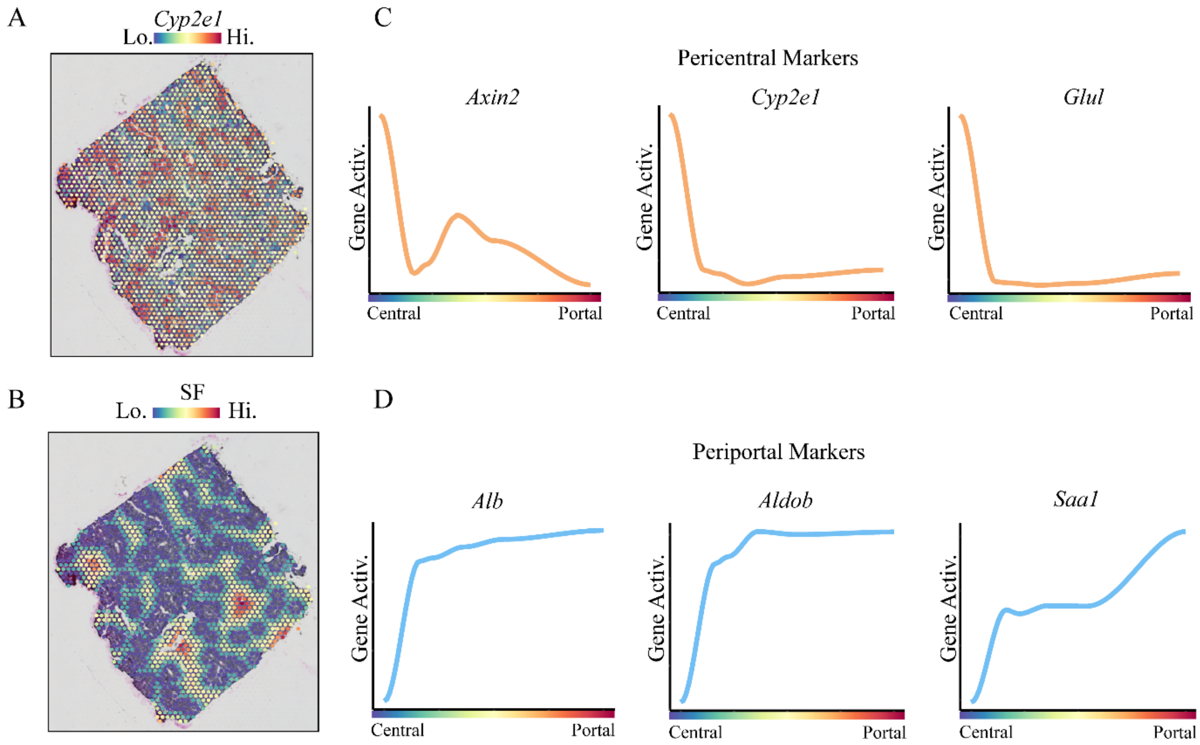

Figure S2 ***SpatialFlux* validation in ST liver zonation dataset**. (**A**) Feature plot showing *Cyp2e1* expression in liver tissue. (**B**) Feature plot showing *SpatialFlux* gradient using as reference points spots highly expressing *Cyp2e1*. (**C**) Curve plots of liver pericentral zonation markers across the distance gradient axis. (**D**) Curve plots of liver periportal zonation markers across the distance gradient axis.
