# Supplementary figures and images for: SpatialFlux: an R package for distance gradient analysis in spatial transcriptomics
Source: Bioinformatics. 2026 Jul 23;42(8):btag543. doi: 10.1093/bioinformatics/btag543 (PMC13430652; doi:10.1093/bioinformatics/btag543)

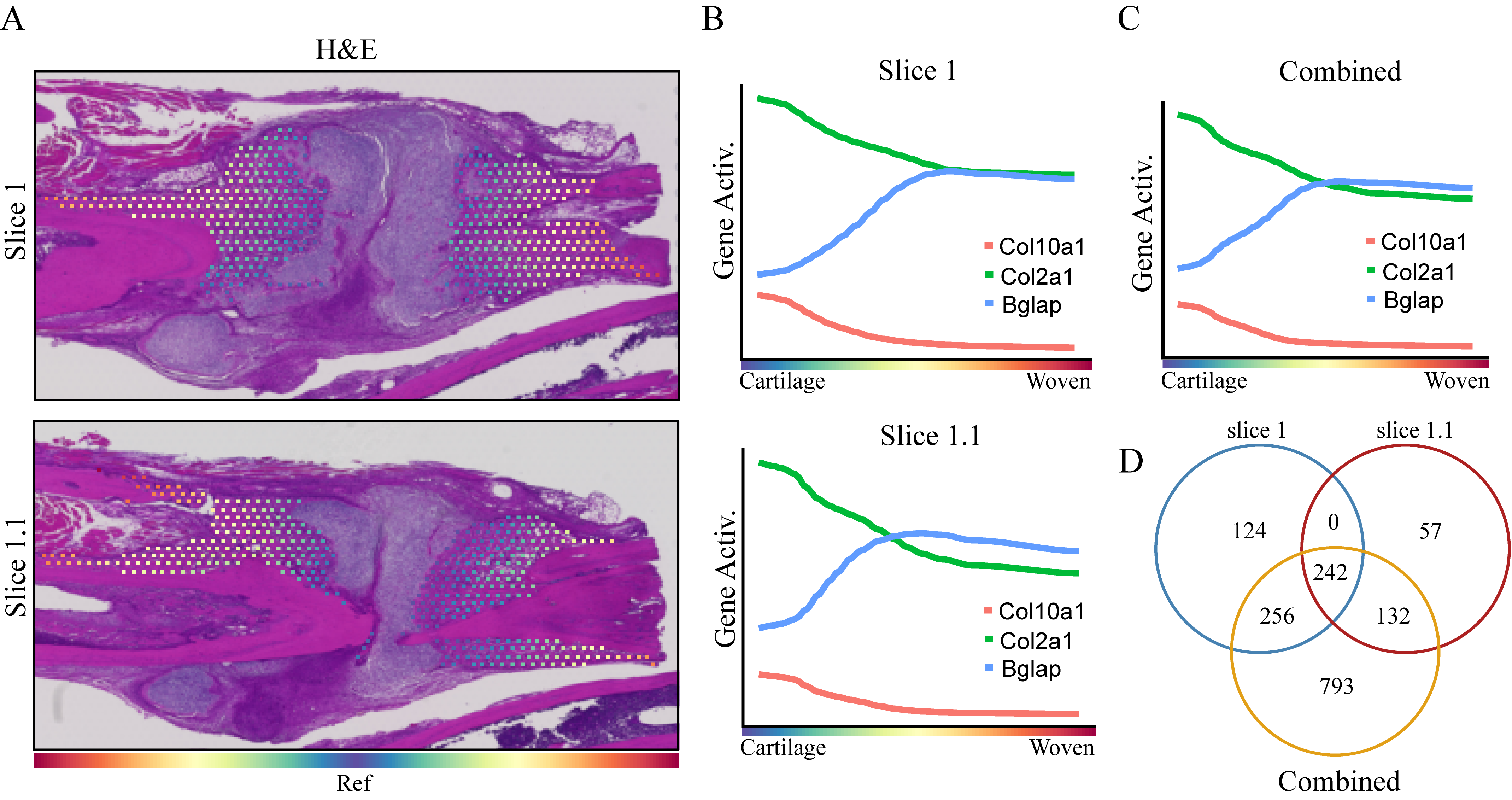

Supplement: btag543_Supplementary_Data [file btag543_supplementary_data.zip › figure S1.tiff]

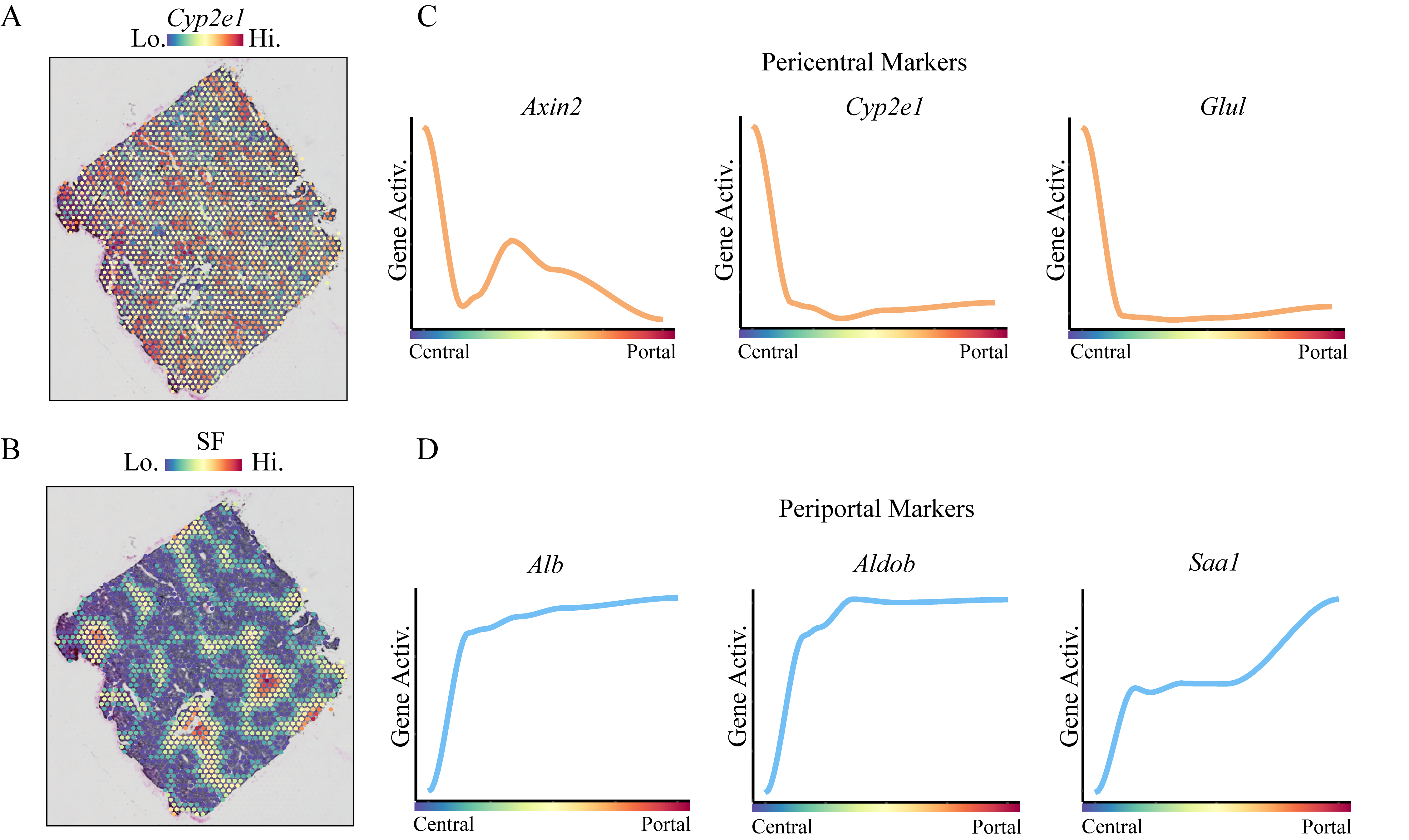

Supplement: btag543_Supplementary_Data [file btag543_supplementary_data.zip › figure s2.tiff]
